# Altered Cortico-Subcortical Network After Adolescent Alcohol Exposure Mediates Behavioral Deficits in Flexible Decision-Making

**DOI:** 10.3389/fphar.2021.778884

**Published:** 2021-11-29

**Authors:** Alexander Gómez-A, Carol A. Dannenhoffer, Amanda Elton, Sung-Ho Lee, Woomi Ban, Yen-Yu Ian Shih, Charlotte A. Boettiger, Donita L. Robinson

**Affiliations:** ^1^ Bowles Center for Alcohol Studies, University of North Carolina, Chapel Hill, NC, United States; ^2^ Department of Psychology and Neuroscience, University of North Carolina, Chapel Hill, NC, United States; ^3^ Biomedical Research Imaging Center, University of North Carolina, Chapel Hill, NC, United States; ^4^ Department of Neurology, University of North Carolina, Chapel Hill, NC, United States; ^5^ Neuroscience Curriculum, University of North Carolina, Chapel Hill, NC, United States; ^6^ Department of Psychiatry, University of North Carolina, Chapel Hill, NC, United States

**Keywords:** adolescence, alcohol, Pavlovian conditioned approach, behavioral flexibility, fMRI

## Abstract

Behavioral flexibility, the ability to modify behavior according to changing conditions, is essential to optimize decision-making. Deficits in behavioral flexibility that persist into adulthood are one consequence of adolescent alcohol exposure, and another is decreased functional connectivity in brain structures involved in decision-making; however, a link between these two outcomes has not been established. We assessed effects of adolescent alcohol and sex on both Pavlovian and instrumental behaviors and resting-state functional connectivity MRI in adult animals to determine associations between behavioral flexibility and resting-state functional connectivity. Alcohol exposure impaired attentional set reversals and decreased functional connectivity among cortical and subcortical regions-of-interest that underlie flexible behavior. Moreover, mediation analyses indicated that adolescent alcohol-induced reductions in functional connectivity within a subnetwork of affected brain regions statistically mediated errors committed during reversal learning. These results provide a novel link between persistent reductions in brain functional connectivity and deficits in behavioral flexibility resulting from adolescent alcohol exposure.

## 1 Introduction

You are heading to dinner at a place near home. Halfway there, it starts raining, and you duck under a shelter. You consider whether you should go back home for an umbrella, wait a few minutes for the rain to stop, or just keep walking to dinner. What do you do? A crucial element of decision-making is flexibility, which implies the ability to adjust behavior in response to both environmental demands and personal factors (Luna, 2009; Diamond, 2013). However, this process requires complementary psychological functions like inhibitory control and working memory, among other components of executive function (Diamond, 2013).

Executive functions recruit subcortical and frontal, parietal and temporal cortical areas (Nowrangi et al., 2014), all of which are still developing during adolescence (Spear, 2013). Nevertheless, there has been a dominant interest in the role of the prefrontal cortex in executive function because of its involvement in “top-down control” (for review see Nowrangi et al., 2014; Dannenhoffer et al., 2018, in press). The prefrontal cortex matures later in development than other cortical regions (Luna, 2009; Spear, 2013), and this process might be altered by recreational drugs (Winters and Arria, 2011), including alcohol (see Spear, 2018; Crews et al., 2019 for review). Since executive function depends on adequate brain development, and flexible decision-making requires proper executive function, any potential hazard to brain development is detrimental to these processes.

Interest in the consequences of underage binge alcohol drinking has increased recently accompanied by many studies in humans and animals models (Dannenhoffer et al., 2021 in press). However, several gaps in knowledge remain, especially for humans, where a lack of longitudinal studies exploring brain circuits and cognitive processing affected by adolescent alcohol exposure is evident. Conversely, investigations in animal models have demonstrated the harmful and persistent effects that adolescent alcohol exposure can have on brain development and, consequently, on behavior. For example, adolescent intermittent ethanol (AIE) exposure can promote adult alcohol intake in male and female rodents (Gass et al., 2014; Amodeo et al., 2017), increase social avoidance (Dannenhoffer et al., 2018), perpetuate adolescent-typical behavior in adulthood (Spear and Swartzwelder, 2014), and impair performance in tasks that require inhibitory control, working memory and flexible decision-making (Schulteis et al., 2008; Salling et al., 2018; Crews et al., 2019; Macht et al., 2020). Moreover, AIE exposure increases conditioned approach to reward-associated cues (McClory and Spear, 2014; Kruse et al., 2017) and impairs flexible responses when experimental conditions change (Coleman et al., 2014; Gass et al., 2014; Fernandez and Savage, 2017; Galaj et al., 2019).

Consistent with those findings, we recently reported that AIE exposure shifted conditioned approach away from goal-tracking and toward sign-tracking during Pavlovian conditioned approach (Madayag et al., 2017). While the goal-tracking response generally adapts to changing conditions, such as reward omission, sign-tracking behavior is less flexible (Morrison et al., 2015; Ahrens et al., 2016; Stringfield et al., 2019). In additional studies, AIE exposure decreased reversal learning of an attentional set (Sey et al., 2019), indicating impaired inhibition of a previously learned response to a reward-predictive cue when the stimulus-response contingency changed. These behavioral tasks are governed by fronto-striatal circuits involved in action selection, and a separate study demonstrated that AIE exposure decreased functional connectivity of these frontostriatal circuits in rats (Broadwater et al., 2018). Together, these findings suggest a potential relationship between inflexible sign-tracking and inflexible attentional set strategy after AIE exposure, possibly mediated by changes in functional connectivity among prefrontal and striatal brain circuits involved in action selection. However, the fact that those results came from separate studies impedes the integration of these AIE effects. Here, we addressed this gap by testing the effects of AIE exposure on sensitivity to conditioned reinforcers (Pavlovian conditioned approach) and behavioral flexibility (attentional set-shifting) within the same animals and integrated them with resting-state functional connectivity MRI among frontal cortical and subcortical regions that regulate action selection. Then, using a dimensionality reduction approach, we identified a principal component of functional connections (functional subnetwork) to test potential mediational effects of functional connectivity on behavior. Thus, through this brain-wide MRI approach, we went beyond the typical analysis of discrete brain regions (i.e., nucleus accumbens, prefrontal cortex, hippocampus) or pairwise connections, which normally use electrochemical, electrophysiological, pharmacological and genetic approaches to understand the neural bases of behavior. Furthermore, the network analysis allows us to propose new circuits and connections that are involved in cognitive processing and decision-making and potentially affected by AIE exposure and to test whether they mediate the observed behavioral deficits.

Based on our previous studies, we hypothesized that AIE exposure promotes greater sensitivity to reward conditioning and inflexible action-selection in adulthood by altering shared underlying neural circuits. Specifically, we predicted that AIE exposure would increase bias to reward-associated cues and impair behavioral flexibility in adulthood when action outcomes change, and that those effects would be mediated by decreased functional connectivity after AIE exposure.

## 2 Methods

### 2.1 Subjects

In this study, we included seventy-nine Sprague-Dawley rats (male *n* = 39, and female *n* = 40), bred and raised in-house. Vivarium temperature and humidity were kept constant with a 12:12 h light cycle applied (lights on at 0700). After birth [postnatal day (P) 1], litters were culled to 8–10 pups with a similar proportion of males and females. As in previous studies, animals were weaned on P21 and pair-housed with a same-sex littermate throughout all testing. Rats received *ad libitum* food and water access with the exception of food-restriction (85% of their free-feeding body weight) beginning 14 days prior to the attentional set-shift task until the fMRI scan was complete. Water restriction required temporary clear dividers between cage mates. All experiments were performed following the NIH Guide for the Care and Use of Laboratory Animals with procedures approved by the Institutional Animal Care and Use Committee of the University of North Carolina at Chapel Hill.

### 2.2 Adolescent Intermittent Ethanol Exposure

**FIGURE 1:**
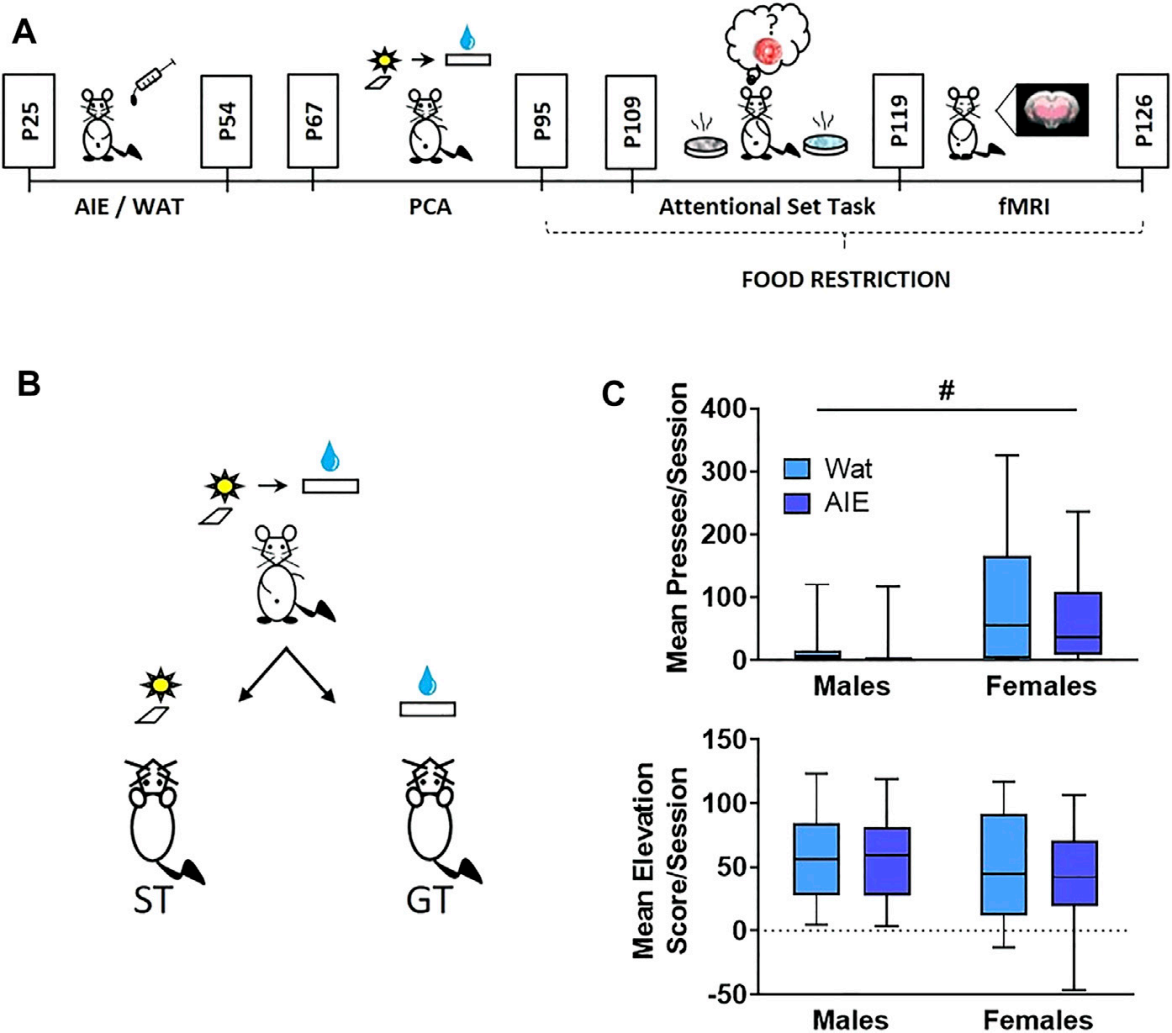
AIE exposure did not affect Pavlovian conditioned approach behavior. **(A)** Scheme illustrating the experimental outline used in this study. Male and female rats were exposed to alcohol (5 g/kg) or water during adolescence. On ∼ P67 rats started behavioral training in Pavlovian conditioned approach (4 weeks) followed by training in an attentional set-shifting task (9 days); the attentional set-shift was preceded by 2 weeks of food restriction. After behavioral training, animals underwent a single session of resting-state fMRI. **(B)** Scheme representing phenotypes of interest in Pavlovian conditioned approach. During training, a compound-cue (light/lever) was presented during a 30-s period, followed by 100 µL of 20% sucrose that served as a reward. Sign-tracking (ST) rats preferentially interacted with the cue (light/lever) while goal-tracking (GT) animals preferentially interacted with the reward receptacle. **(C)** Top: Sex, but not AIE exposure, promoted ST behavior, reflected in more conditioned lever presses by female rats than males. Bottom: No differences were observed in elevation score, a measure of GT behavior. Since the data were not normally distributed, data are presented in box plots showing median (horizontal line), interquartile range (box), and minimum and maximum data values (lower and upper whiskers). # main effect of sex. Detailed statistical analyses are provided in Table 1, additional data from Pavlovian conditioned approach are shown in Supplementary Figure S1.

Starting at P25, animals were weighed and given 5 g/kg of ethanol (25% v/v in water) or an equivalent volume of water (controls) intragastrically, in an intermittent pattern (once/day on a 2-days-on, 2-days-off schedule) until P54 for a total of 16 exposures (Figure 1A). This dose produces blood alcohol levels of approximately 230 mg/dl in males and females 60 min after administration (Madayag et al., 2017). Animals housed together were given the same exposure (either alcohol or water) throughout adolescence. Rats from a litter were distributed across all experimental groups whenever possible in a 2 (sex) x 2 (exposure) factorial design.

### 2.3 Pavlovian Conditioned Approach

Operant chambers (Med Associates, St. Albans, VT) were used to train animals in Pavlovian conditioned approach (Madayag et al., 2017). At ∼ P70, rats were given home-cage access to 20% sucrose in water for 1 h prior to magazine training to become familiarized with the reward. After that, animals were placed in behavioral chambers (12″ length × 12.5″ width × 11.5″ height) for receptacle training to familiarize them with sucrose delivery every 120–230 s into the receptacle. After the session, the receptacle was checked to verify that the animal consumed the reward. Next, Pavlovian conditioning occurred daily for 20 days across 4 weeks (Figure 1A). Rats were presented with a conditioned stimulus (CS; illuminated cue light plus lever extension) for 30 s. At the end of the 30-s period, the lever retracted, the light turned off, and a reward (100 µL 20% sucrose in water) was delivered into the receptacle. Fifteen trials occurred throughout the session with a variable inter-trial interval (90–210 s). Behavioral measures (lever presses, receptacle entries) and conditioning events (CS + onset and offset, sucrose delivery) were recorded and timestamped by Med Associates for later analysis.

We monitored both lever presses and receptacle entries, and calculated the latency to press the lever or enter the receptacle after the onset of the CS. The probability to press the lever or enter the receptacle during the CS was measured as the number of trials that each behavior occurred divided by 15 (the total number of trials). For receptacle entries, we calculated an elevation score, measured as the number of receptacle entries during a CS presentation less the number of receptacle entries 30 s prior; this described those entries that were conditioned responses to the CS (Besheer et al., 2004). For data analysis, we averaged metrics of conditioned approach across the five last sessions to assess effects of sex or alcohol exposure. Behaviors were categorized as either sign-tracking (lever-directed) or goal-tracking (receptacle-directed; Figure 1B).

### 2.4 Attentional Set-Shift Task

Discriminative association was measured using a digging attentional set task (Birrell and Brown, 2000; Sey et al., 2019), wherein a reward (1/4 of a fruit loop cereal) was buried in digging media and associated with a specific odor (e.g., vanilla) placed in one of two dishes (Figure 1A; see Supplementary Table S1 for a detailed description). Animals were required to perform six correct choices in a row to reach criterion and advance to the next step.

The attentional set-shift was preceded by 2 weeks of food restriction. Animals were maintained at 85% of their baseline weight with water ad libitum. During the training phases, animals received food in the home cage after task completion.

On training days 1–3, rats learned to retrieve a reward from one of two dishes at the end of the chamber unencumbered by digging media at first. On days 4–5, dishes contained digging media (white or brown bedding) and the rat learned to dig to find and consume the reward.

Acquisition of the discriminative attentional set began on day 6. Randomizing the digging media and side of the box, the reward was placed in a dish with a corresponding odor (e.g., vanilla) while the other dish (e.g., cinnamon) did not contain a reward. On each trial, the rat chose a dish by digging with the face or paws; at that point, entry to the other dish was blocked. If correct, the rat was given time to consume the reward. If incorrect, the rat was given time to realize no reward was present. If the rat did not make a choice within a 3-min period, the trial was recorded as an omission and counted against their criterion. When the rat chose the correct dish six consecutive times (Birrell and Brown, 2000; Sey et al., 2019), the rat reached criterion and finished testing for that day.

On the first reversal day (Reversal 1, day 7), the discrimination task began with reacquisition of the association from the previous day. Once the rat reached criterion for reacquisition (6 consecutive correct choices), the contingency was reversed such that the opposite odor (e.g., cinnamon) signified the reward location. Rats continued testing until criterion was met.

A second reversal was conducted the next day (Reversal 2, day 8) wherein the association was switched back to the original contingency used on day 6 after reacquisition was completed. Rats once again continued testing until criterion was met and data for all trials were recorded.

The following day (Extradimensional Set-Shift, day 9), after the rat completed reacquisition, there was an extradimensional set-shift of sensory modality of the discriminant using novel stimuli. If vanilla and cinnamon were the odors previously used, the set-shift used coconut and paprika. Likewise, if white and brown crinkle paper were previously used, the set-shift used gravel and sand. Importantly, the discriminant associated with the reward was now the digging medium and odor was irrelevant.

Errors after a contingency reversal were categorized as prepotent or regressive. Upon reversal, errors made prior to a correct choice within the new contingency were recorded as prepotent responses. Once a correct choice had been made, and a reward was reached (positive feedback), all future errors in that contingency were recorded as regressive errors. Regressive errors were further divided according to whether it occurred after a correct choice (initial regressive error) or after an error itself (subsequent regressive error). As mentioned in the results, initial and subsequent regressive errors reflect distinct deficits: initial regressive errors indicate difficulties in performing a behavior based on the positive feedback after adequate discrimination, while subsequent regressive errors suggest deficits in behavior modification based on feedback, whether positive or negative. This difference provides information on how much the cues and the presence or absence of the reward control behavior, as well as the cognitive processes required to maintain information about relevant contingencies.

### 2.5 Resting State fMRI

#### 2.5.1 Animal Preparation

The MRI component of the study was performed on a subset of 41 rats (male *n* = 19, and female *n* = 22) from the larger sample that underwent the Pavlovian conditioned approach and set-shifting task (Figure 1A). Although the initial plan was to include the same number of animals in behavioral training and MRI scans, technical issues with the MRI scanner obliged us to keep training animals until the problem was resolved. Thus, whether animals underwent scans was based solely on scanner availability. The endotracheal tube (Surflash Polyurethane IV Catheter 14G x 2″, TERUMO, Somerset, NJ, United States) was intubated immediately after anesthetic induction with 4% isoflurane to conduct mechanical ventilation at 60 breaths/min with an inspiration time at 40% ratio using MRI-Compatible Ventilator (MRI-1, CWE Inc., Ardmore, PA, United States). The animals were anesthetized under 2% isoflurane (Isoflurane Vaporizer #911103, VetEquip Inc., Livermore, CA, United States) during positioning on a custom-built cradle. MR compatible sensors were installed to monitor core body temperature (OAKTON Temp9500, Cole-Parmer, Vernon Hills, IL, United States), heart rate, peripheral blood oxygen saturation, and end-tidal CO_2_ (SURGIVET® V90041LF, Smith Medical, Dublin, OH, United States). The body temperature was maintained at 37 ± 0.5°C using a circulating water blanket connected to a temperature adjustable water bath (Haake S13, Thermo Fisher Scientific, Waltham, MA, United States). The tidal volume of mechanical ventilation was adjusted to keep the heart rate at 300 ± 50 beats per minute, peripheral blood oxygen saturation above 96%, and end-tidal CO2 between 2.8–3.2%. To reliably probe functional connectivity, a continuous infusion of dexmedetomidine (0.05 mg/kg/hr) and pancuronium bromide (0.5 mg/kg/hr) was initiated 30 min before the MRI scans and isoflurane was reduced to 0.5% before fMRI experiments (Broadwater et al., 2018).

#### 2.5.2 fMRI Acquisition

All MR images were conducted on a Bruker BioSpec 9.4-T, 30 cm bore system. A 72 mm volume transmitter coil and a 4-channel receiver array coil were used. Magnetic field homogeneity was optimized first by global shimming, followed by local second-order shims using a MAPSHIM map protocol. We acquired a single 30 min BOLD fMRI scan using a 2D multi-slice, single-shot, gradient-echo, echo-planar imaging (EPI) sequence [scan parameters: TR (repetition time) = 2000 ms, TE (echo time) = 14 ms, bandwidth = 250kHz, flip angle = 70°, voxel size = 0.4 × 0.4 × 0.4 mm^3^, matrix size = 72 × 72 × 32, and FOV (field of view) = 28.8 × 28.8 × 12.8 mm^3^].

#### 2.5.3 Data Preprocessing

Raw scan data were downloaded from the Bruker 9.4T scanner, converted into NifTI-1 data format, and organized based on the Brain Imaging Data Structure (BIDS) guideline using an in-house built converter. The data preprocessing was initiated with the slice timing correction and rigid body motion correction using the command-line interfaces of Analysis of Functional Neuroimages (AFNI) software (Cox, 1996). Manual skull stripping was performed on the first frame of the functional connectivity MRI image to precisely employ non-linear spatial normalization using the Symmetric Normalization (SyN) algorithm of the advanced normalization tools (ANTs) package (Avants et al., 2008). The EPI-based brain template was used as a reference for spatial normalization. Finally, we performed signal processing on a time-domain signal using SciPy (Virtanen et al., 2020) python library. These signal processes include nuisance signal regression with six rigid motion parameters, bandpass filtering at 0.01–0.15 Hz, and spatial smoothing using a Gaussian kernel function with a full width at half maximum (FWHM) expression extent at 0.5 mm. Our automated preprocessing pipeline was built using Python Neuroimaging Pipeline Tool (PyNIPT), an open-source pipeline framework, and available at GitHub repository (https://github.com/CAMRIatUNC).

### 2.6 Data Analysis

#### 2.6.1 Behavior

Statistical analyses of behavioral data were conducted using SPSS for Microsoft (version 26, available from the Virtual Lab of the University of North Carolina at Chapel Hill). Most behavioral data were not normally distributed; thus, the effects of AIE exposure and sex on behavioral metrics from conditioned approach and the attentional set-shifting task were analyzed using a generalized linear model with a Poisson distribution and a log link function. Post-hoc comparisons were Bonferroni-corrected. The elevation score (normally distributed) was analyzed using a two-way ANOVA in GraphPad (version 8, San Diego, CA). Statistical significance was set at *p* ≤ 0.05 and marginal significance was set at *p* ≤ 0.10.

#### 2.6.2 MRI ROI Analysis

We determined eight ROIs: the prelimbic prefrontal cortex (PrL), infralimbic cortex (IL), nucleus accumbens (NAc), caudate putamen (CPu), orbitofrontal cortex (OFC), dorsal hippocampus (HippD), thalamus (Thal), and primary somatosensory cortex (S1). All ROIs were manually drawn on a T2-weighted MRI template co-registered to the Paxinos & Watson rat brain atlas (Paxinos and Watson, 2007), then warped to the matching EPI MRI templates to ensure that all ROIs were aligned in the correct position within the brain in the fMRI data. The resting-state BOLD signal in each ROI was extracted from the preprocessed EPI data that had been aligned to this EPI MRI template and averaged to use for correlation analysis. Pearson’s correlation coefficients were computed in pairs between ROIs to create a connectivity matrix at the individual level. Fisher’s transform was applied to convert the correlation coefficient into approximately normal distribution (Fisher’s z score) for group statistics. Two-way ANOVA was conducted with 2 × 2 between-subject (sex and alcohol exposure) design using statsmodels (Seabold and Perktold, 2010), a Python module for statistical modeling. To identify significant changes in connectivity, the suprathreshold of *p* < 0.05 was applied to build sparse matrix for each subject, and we controlled for multiple comparisons by employing link-based family-wise error rate using the network-based statistics (Zalesky et al., 2010) approach. Significant changes in connectivity in pairwise ROIs were determined by thresholding at a corrected p-value < 0.05.

#### 2.6.3 Correlational Analyses

To assess the statistical correlation between behavior and fMRI, we calculated the Pearson’s correlation coefficient between behavior scores and the functional connectivity for each pair of regions. The p values were estimated based on the beta distribution on the interval from −1 to 1 with equal shape parameters a = b = n/2–1. Subsequently, a false discovery rate correction was applied to control for multiple comparisons. Like the mediational analysis, we focused on active errors during acquisition in Reversal 2 as these showed robust behavioral effects of AIE exposure. Statistical significance was set at a corrected *p* < 0.05.

#### 2.6.4 Mediation Analyses

To test the hypothesis that the effects of AIE exposure on behavior (i.e., AIE → behavior) were mediated by AIE-related changes in brain functional connectivity (i.e., AIE → brain functional connectivity → behavior), we conducted a causal mediation analysis. Causal mediation is a method of assessing the directed or causal relationships among variables (MacKinnon et al., 2007; Yung et al., 2018). Due to the large number of pairwise brain connections that could serve as potential mediators, the high correlation among ROIs, and the consistent negative direction of AIE exposure effects on brain connectivity, we used a dimensionality reduction approach to calculate a single measure of brain functional connectivity. First, we selected an informative set of pairwise connections which varied with AIE exposure at a lenient statistical threshold of *p* < 0.1, uncorrected (*n* = 11; Figure 7A). This somewhat lenient threshold was chosen since connections with weaker associations in univariate analyses can still contribute meaningful variance when combined with other variables (Chowdhury and Turin, 2020). Next, we conducted a principal component analysis of this reduced functional connectivity matrix, which yielded a first principal component accounting for 66% of the variance in functional connectivity among the included connections. The positive component weights for each connection were consistent with the interpretation of the set of connections as a cohesive functional subnetwork of the included ROIs. The component score for each subject was entered as the brain functional connectivity mediator in the mediation analysis.

Mediation analyses were conducted in SAS 9.4 using the CAUSALMED Procedure (Yung et al., 2018) using a counterfactual framework (Robins and Greenland, 1992), which allows for testing of nonlinear effects such as count data, for active errors during acquisition in Reversal 2 that demonstrated consistent AIE exposure effects in both the MRI subsample and the larger sample (see Table 1 and Supplementary Figure S5). The effect of AIE exposure on brain functional connectivity was modeled with a normal distribution, whereas the effects of AIE exposure and brain functional connectivity on the behavioral variables were modeled with a Poisson distribution using a log link function. Sex was included as a covariate.

**TABLE 1:** Statistical analysis results of the Pavlovian conditioned approach and the attentional set-shifting task (*n* = 79 rats).

I. Pavlovian Conditioned Approach					
**Variable**	**Effect**	***p***	**Exp (B)**	**Coefficient interval**	
				**lower upper**	
Lever presses	Exposure	0.39	0.508	0.105	2.453
	Sex	<0.01*	4.541	1.697	12.148
	Exp. x Sex	0.70	1.387	0.251	7.66
Lever latency	Exposure	0.27	1.110	0.920	1.338
	Sex	<0.01*	0.721	0.590	0.883
	Exp. x Sex	0.75	0.954	0.714	1.275
Lever probability	Exposure	0.09	0.509	0.228	1.136
	Sex	<0.01*	2.274	1.322	3.912
	Exp. x Sex	0.23	1.743	0.695	4.367
Receptacle entries	Exposure	0.67	0.931	0.664	1.305
	Sex	0.59	1.088	0.795	1.490
	Exp. x Sex	0.81	0.944	0.590	1.512
Receptacle latency	Exposure	0.96	0.993	0.736	1.339
	Sex	0.09	0.769	0.565	1.045
	Exp. x Sex	0.48	1.171	0.753	1.82
Receptacle probability	Exposure	0.62	0.978	0.895	1.068
	Sex	0.14	1.064	0.979	1.157
	Exp. x Sex	0.73	0.979	0.866	1.108
**Variable**	**Effect**	**p**	**F**	**Df**	**—**
Elevation Score^@^	Exposure	0.41	0.658	1	—
	Sex	0.19	1.673	1	
	Exp. x Sex	0.57	0.321	1	

II. Attentional Set-Shift Task					
**a. Acquisition**					
**Variable**	**Effect**	**p**	**Exp (B)**	**Coefficient Interval**	
				**Lower Upper**	
Total trials acquisition	Exposure	0.92	0.984	0.725	1.336
	Sex	0.26	1.174	0.885	1.558
	Exp. x Sex	0.58	0.890	0.584	1.356
Active errors acquisition	Exposure	0.26	0.622	0.270	1.434
	Sex	0.43	1.302	0.671	2.527
	Exp. x Sex	0.83	1.123	0.376	3.35
**b. Reversal 1**					
Total trials reacquisition	Exposure	0.65	0.951	0.761	1.188
	Sex	0.30	0.893	0.718	1.110
	Exp. x Sex	0.26	1.198	0.875	1.642
Active errors reacquisition	Exposure	0.33	0.652	0.275	1.545
	Sex	0.11	0.476	0.192	1.184
	Exp. x Sex	0.05*	3.581	1.021	12.557
Total trials acquisition (R1)	Exposure	0.66	1.066	0.799	1.422
	Sex	0.57	0.921	0.690	1.228
	Exp. x Sex	0.55	1.132	0.753	1.701
Active errors acquisition (R1)	Exposure	0.25	1.173	0.891	1.545
	Sex	0.42	0.890	0.670	1.183
	Exp. x Sex	0.14	1.330	0.904	1.956
Prepotent errors (R1)	Exposure	0.08	1.525	0.950	2.447
	Sex	0.80	1.066	0.649	1.749
	Exp. x Sex	0.39	1.320	0.699	2.496
Regressive errors (R1)	Exposure	0.95	1.021	0.532	1.960
	Sex	0.54	0.814	0.418	1.584
	Exp. x Sex	0.60	1.277	0.5	3.258
Initial regressive errors (R1)	Exposure	0.86	1.053	0.591	1.876
	Sex	0.46	0.785	0.431	1.431
	Exp. x Sex	0.59	1.253	0.541	2.903
Subsequent regressive errors (R1)	Exposure	0.93	0.961	0.376	2.458
	Sex	0.76	0.870	0.344	2.199
	Exp. x Sex	0.67	1.329	0.355	4.977
**c. Reversal 2**					
Total trials reacquisition	Exposure	0.08	1.197	0.977	1.467
	Sex	0.98	0.997	0.813	1.224
	Exp. x Sex	0.89	1.080	0.813	1.433
Active errors reacquisition	Exposure	0.03*	1.905	1.061	3.421
	Sex	0.34	1.342	0.73	2.466
	Exp. x Sex	0.94	0.973	0.451	2.098
Total trials acquisition (R2)	Exposure	0.10	1.060	0.757	1.485
	Sex	0.04*	1.110	0.805	1.532
	Exp. x Sex	0.26	1.298	0.828	2.034
Active errors acquisition (R2)	Exposure	<0.01*	1.563	1.144	2.135
	Sex	0.10	1.295	0.946	1.774
	Exp. x Sex	0.46	1.165	0.775	1.750
Prepotent errors (R2)	Exposure	0.02*	1.842	1.116	3.042
	Sex	0.06	0.530	0.274	1.025
	Exp. x Sex	0.76	1.137	0.497	2.604
Regressive errors (R2)	Exposure	0.10	1.404	0.941	2.094
	Sex	<0.01*	1.753	1.208	2.545
	Exp. x Sex	0.37	1.247	0.764	2.037
Initial regressive errors (R2)	Exposure	0.60	1.18	0.633	2.202
	Sex	0.22	1.433	0.802	2.558
	Exp. x Sex	0.36	1.434	0.655	3.139
Subsequent regressive errors (R2)	Exposure	0.05*	2.222	1.005	4.912
	Sex	<0.01*	2.828	1.335	5.994
	Exp. x Sex	0.75	0.864	0.343	2.177
**d. Set shift**					
Total trials reacquisition	Exposure	0.35	0.871	0.649	1.168
	Sex	0.50	0.909	0.687	1.202
	Exp. x Sex	0.56	1.131	0.745	1.716
Active errors reacquisition	Exposure	0.44	0.741	0.343	1.6
	Sex	0.97	1.010	0.511	1.998
	Exp. x Sex	0.66	1.265	0.443	3.613
Total trials acquisition (SS)	Exposure	0.88	1.017	0.804	1.287
	Sex	0.97	0.997	0.793	1.251
	Exp. x Sex	0.30	1.186	0.857	1.64
Active errors acquisition (SS)	Exposure	0.42	1.203	0.762	1.899
	Sex	0.77	0.935	0.586	1.493
	Exp. x Sex	0.16	1.552	0.833	2.891

AIE, exposure and sex effects on conditioned approach (except elevation score) and the attentional set-shifting task metrics were analyzed using a generalized linear model with a Poisson distribution and a log link function. The elevation score (@) analysis was carried out using a two-way ANOVA. * Significant at *p* ≤ 0.05. Bold type indicates *p* ≤ 0.05.

## 3 Results

### 3.1 Pavlovian Conditioned Approach

Animals trained on Pavlovian conditioned approach for 20 daily sessions across 4 weeks, in which a 30-s stimulus (light and lever extension) predicted a sucrose reward. Lever-directed behaviors included the number of lever presses, latency to press the lever within trials, and probability of pressing the lever within sessions. Receptacle-directed behaviors included the receptacle entry elevation score, latency to enter the receptacle, and probability of entering the receptacle. Using a generalized linear model and calculating odds ratios (OR) and their corresponding 95% confidence intervals (CI; Methods), we found significant main effects of sex across all sign-tracking behaviors, with females pressing the lever more times, sooner, and with higher probability of any lever press (Figure 1C; Supplementary Figure S1A; full statistical outcomes in Table 1). We also observed that AIE-exposed rats were marginally less likely to press the lever than controls (lever press probability, *p* = 0.09). No interaction between exposure and sex was observed for any lever-directed metric (all *p* > 0.05; Table 1). Regarding goal-tracking behaviors, female rats entered the receptacle marginally faster than males (*p* = 0.09). However, no significant effects of AIE exposure, sex or exposure-by-sex interaction were found for receptacle-directed behavioral metrics (all *p* > 0.05; Figure 1C; Supplementary Figure S1B; Table 1). In summary, our data found that females were biased to interact with the cue, independent of adolescent alcohol exposure.

### 3.2 Attentional Set-Shifting Task

**FIGURE 2:**
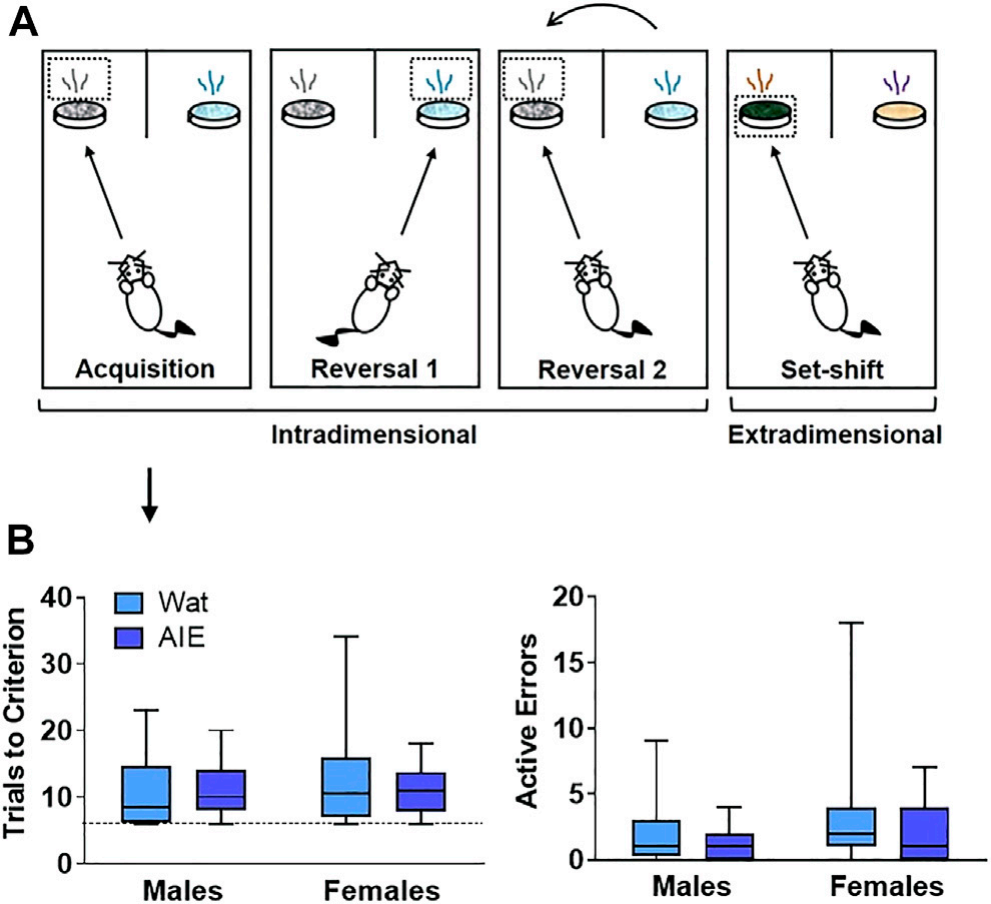
AIE exposure did not impair initial discriminative learning in an attentional set-shifting task. **(A)** Scheme of the attentional set-shifting task. We trained rats to discriminate between two different odors, one of which was associated with a food reward (details about task in Supplementary Table S1). Cups containing rewards were covered with digging media, and animals were trained to dig inside the container according to the odor predicting the food, independent of the digging medium covering the cups. After initial training, intradimensional reversals 1 and 2 were introduced, keeping odor as the relevant dimension. Lastly, the extradimensional set-shift phase was initiated with novel stimuli, when the appropriate discriminant was the medium instead of odor. Criterion was set at six consecutive correct choices. **(B)** Groups did not differ in the total number of trials that animals required to reach criterion or the number of errors made. Arrow indicates that graphs in panel B describe acquisition phase. Box plots represent median, interquartile range and minimum and maximum data values. Detailed statistical analyses are provided in Table 1.

Next, we trained rats on an attentional set-shifting task (Birrell and Brown, 2000) (Figures 1A, 2A). We measured the number of trials that animals required to reach the criterion of six consecutive correct choices, as well as the total number and type of errors made, during each phase of the task: Acquisition, Reversal 1, Reversal 2, and Extradimensional Set-Shift (full statistics in Table 1).

#### 3.2.1 Acquisition of the Compound Stimulus Discrimination

Neither AIE exposure nor sex affected the total number of trials that animals required to reach criterion, or the number of errors made. Moreover, no interaction was observed for any parameter (all *p*’s > 0.05). Thus, the process of learning an initial compound discrimination was unaffected by AIE exposure or sex.

#### 3.2.2 Reversal 1

**FIGURE 3:**
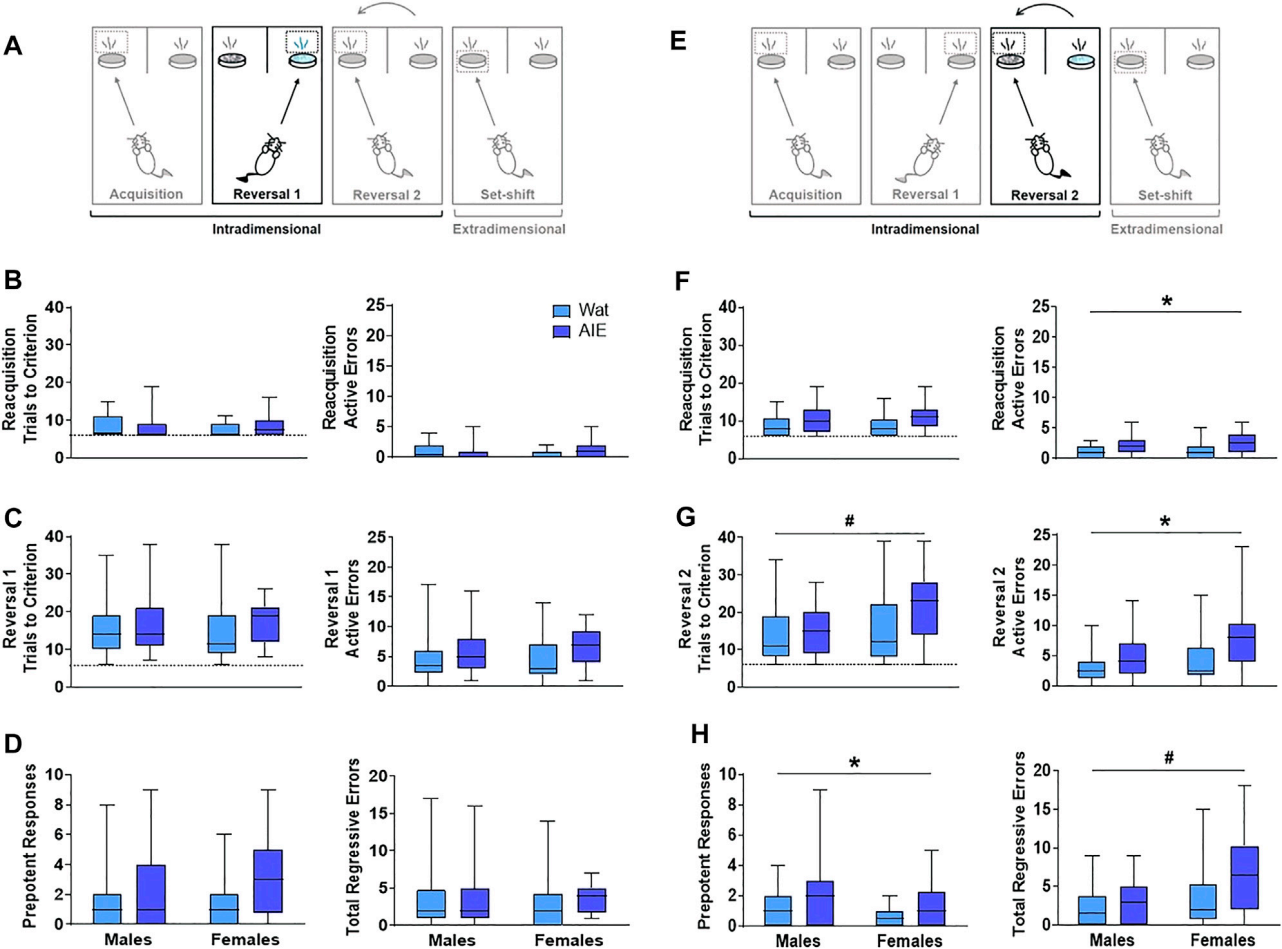
AIE exposure impaired some parameters in reversal learning but not acquisition of rules. **(A)** On Reversal 1, the contingency was reversed such that the opposite odor signified the reward location. **(B)** Every new training day began with reacquisition of the association from the previous day (reacquisition). Groups did not differ in the number of trials to reach criterion **(left)**. We observed a significant interacting effect of exposure and sex on active errors during reacquisition **(right)**; however, no group differences survived Bonferroni correction. **(C)** During Reversal 1, groups did not differ in the total number of trials required to reach criterion **(left)** or the number of active errors while reaching criterion **(right)**. **(D)** As active errors entail different types of errors, we subdivided them into “prepotent” and “regressive,” depending on whether they occurred before or after a correct response, respectively. Groups did not significantly differ in the number of prepotent **(left)** or regressive **(right)** errors. **(E)** The next day, rats underwent reacquisition followed by Reversal 2, wherein the association was switched back to the original contingency used on Acquisition. **(F)** In reacquisition of the previous day’s rule, AIE-exposed animals committed more errors than control rats **(right)**, suggesting that AIE exposure impaired consolidation of what animals learned the day before. **(G)** When Reversal 2 was introduced, female rats required more trials than male rats to reach criterion **(left)**. However, AIE-exposed animals committed more total errors than water-exposed rats **(right)**. **(H)** Regarding error type, AIE exposure was associated with more prepotent errors **(left)**, while female sex was associated with more regressive errors **(right)**. Statistics and figures for initial regressive and subsequent errors are showed in Supplementary Figure S2. AIE-exposed animals had more difficulty inhibiting learned rules, updating learned information, and guiding behavioral choices based on feedback than water-exposed animals, effects that were generally larger in females. Box plots show median, interquartile range and minimum and maximum data values. # main effect of sex (*p* < 0.05). * main effect of exposure (*p* < 0.05). Detailed statistical analyses are provided in Table 1.

From this point, each phase started with the discriminations learned the previous day (reacquisition) before introducing the new rule (reversal; Figure 3A). No differences were observed in the trials to reach criterion for reacquisition as a function of exposure or sex, and no interaction between factors was identified (all *p*’s > 0.05; Figure 3B). However, we observed a significant exposure-by-sex interaction on the number of active errors during reacquisition (*p* = 0.05; Figure 3B). AIE-exposed females exhibited more active errors during reacquisition than controls; however, pairwise differences did not survive Bonferroni correction in post-hoc comparisons.

After rats obtained reacquisition criterion, the contingency of the olfactory cues was switched (Reversal 1). Neither the total number of trials required to reach criterion nor the number of errors committed differed across groups; no main effects of exposure or sex and no exposure-by-sex interaction reached significance (all *p*’s > 0.05; Figure 3C and Table 1). To better understand the type of errors animals made during their performance, we subdivided errors into “prepotent” and “regressive,” depending on whether they occurred before or after a correct response, respectively, with regressive errors being further subdivided into initial and subsequent. We observed a trending increase (∼50%) in prepotent responses in AIE-exposed animals compared to controls (*p* = 0.08; Figure 3D), with no main effect of sex or exposure-by-sex interaction (all *p*’s > 0.05, Table 1). As prepotent responses occurred until the first correct response in the new rule, one might expect that positive feedback from reinforcement under the new rule would improve animals’ performance. In fact, that was what we observed during Reversal 1, as regressive errors were similar among groups (Figure 3D), as were subtypes of regressive errors (Table 1). These results suggest that AIE-exposed animals had some difficulty switching from the previously learned rule as compared to water-exposed siblings. However, after positive feedback, learning of the new rule was similar among groups.

#### 3.2.3 Reversal 2

At this stage, both odors had been associated with reward; thus, performance here does not imply new learning. Instead, animals must update their attentional set to a previously learned rule (Figure 3E). In the reacquisition phase, AIE-exposed animals needed marginally more trials to reach criterion (*p* = 0.08) and committed more errors (*p* = 0.03; Figure 3F) than controls. There were no sex differences or exposure-by-sex interactions (all *p*’s > 0.05; Table1), suggesting that AIE exposure impaired consolidation of the contingency the animals learned the day before.

After reacquisition, the original discrimination rule from Day 6 was re-introduced (Reversal 2). AIE- and water-exposed rats did not differ in the number of trials to reach criterion (Table 1); however, female rats required more trials than did males (*p* = 0.04; Figure 3G). No exposure-by-sex interaction was observed (*p* > 0.05). In analyzing the errors committed during Reversal 2, we found that AIE-exposed animals made more total errors than water-exposed rats (*p* = 0.01; Figure 3G and Table 1), and female rats showed a marginal increase in total errors compared to males (*p* = 0.10), but no exposure-by-sex interaction was observed (*p* > 0.05). Regarding error type, both AIE exposure (*p* = 0.02) and, marginally, female sex (*p* = 0.06; Figure 3H and Table 1) were associated with more prepotent errors, but no exposure-by-sex interaction was observed (Table 1). Similarly, total regressive errors were marginally higher in AIE-exposed animals (*p* = 0.10), and significantly higher in female rats (*p* < 0.01; Figure 3H), with no exposure-by-sex interaction (Table 1). Considering that Reversal 2 involves an update of previous learning, these results indicate that AIE-exposed animals had difficulty either recovering the original rule or keeping track of the current rule, and the positive feedback after a correct response did not maintain performance as it did in Reversal 1.

We further categorized regressive errors as initial and subsequent regressive errors. Initial regressive errors were not different between exposure or sex (all *p*’s > 0.05, Supplementary Figure S2A, Table 1). However, AIE-exposed animals made more subsequent regressive errors than water-exposed animals (*p* = 0.05) and females made more subsequent regressive errors than males (*p* = 0.01, Supplementary Figure S2B, Table 1). No exposure-by-sex interaction was identified for either initial or subsequent regressive errors (all *p*’s > 0.05). Together, these results suggest that ethanol exposure during adolescence impairs the ability to update information about learned strategies and the capacity to use feedback to guide behavioral choice.

#### 3.2.4 Extradimensional Set-Shift

**FIGURE 4:**
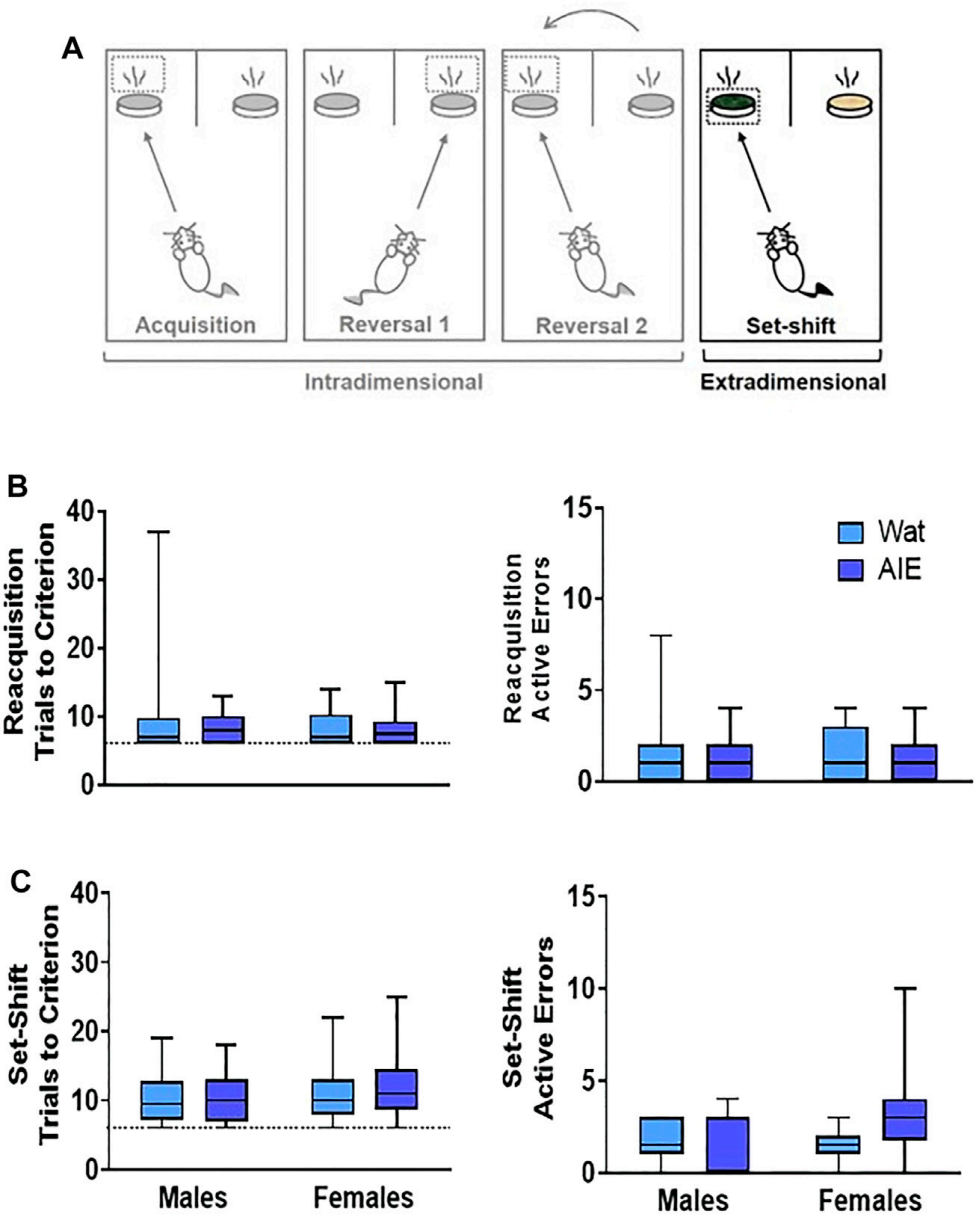
AIE exposure did not impair extradimensional set-shifting performance. **(A)** After reversals, an extradimensional set shift to completely new stimuli and sensory modality of the discriminant was introduced. In this phase, the discriminant associated with the reward was now the digging medium and odor was irrelevant. **(B)** In the reacquisition phase, groups did not differ in the total trials to reach criterion **(left)** or active errors **(right)**. **(C)** In the extradimensional set shift, groups did not differ in the total trials required to reach criterion **(left)** or the number of active errors made **(right)**, suggesting that AIE exposure does alter the ability to shift sensory modality of the discriminant in the context of new stimuli. Box plots show median, interquartile range and minimum and maximum data values. Detailed statistical analyses are provided in Table 1.

The reacquisition phase resulted in no differences or interactions were observed in the total trials to reach criterion or active errors (all *p*’s > 0.05, Table 1). Next, the extradimensional set-shift began with the new set of stimuli as well as new relevant sensory dimension (media rather than odor). Similar to the first Acquisition Day, total trials required to reach criterion and active errors were not affected (all *p*’s > 0.05; Figure 4C and Table 1). These results indicate that, despite a change in sensory modality of the relevant stimulus, discrimination learning to novel stimuli was unaltered after AIE exposure, consistent with results observed in other studies (Gass et al., 2014; Sey et al., 2019).

### 3.3 Resting State fMRI

**FIGURE 5:**
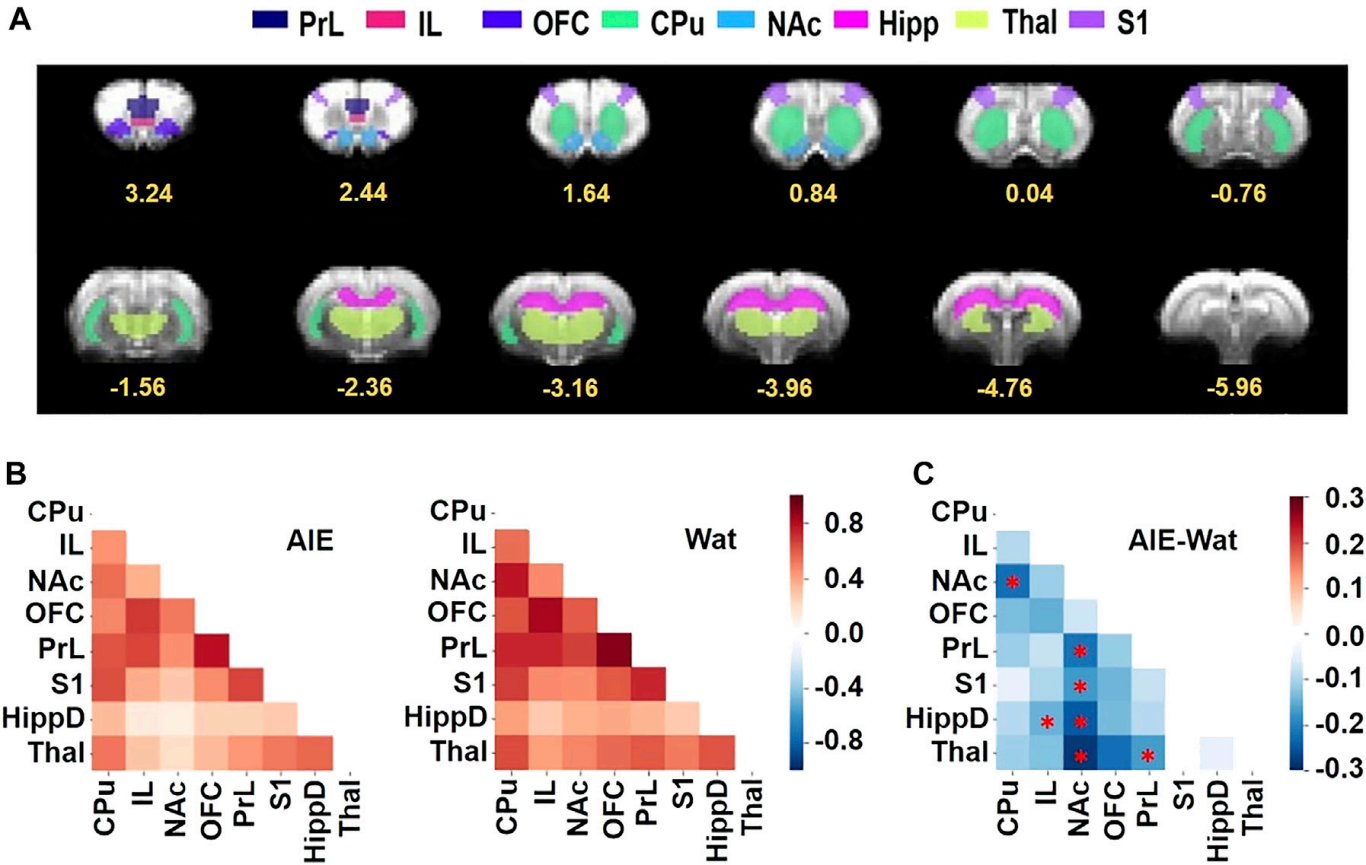
AIE exposure decreased resting-state functional connectivity among regions of interest (ROIs) involved in behavioral flexibility. **(A)** ROIs used for analyses shown on the EPI template were manually drawn by co-registering the T2 template to the Paxinos & Watson rat brain atlas (Paxinos and Watson, 2007), then aligned to the matching EPI templates to ensure that all ROIs were aligned in the correct position within the brain in the fMRI data. Numbers below slices indicate location in mm from bregma. **(B)** The Z-score correlation matrixes for the AIE **(left)** and Wat control **(right)** groups describe the functional connectivity among ROI pairs, with warm colors (positive Z scores) indicating increased connectivity and cool colors (negative Z scores) indicating decreased connectivity. **(C)** AIE-exposed rats exhibited less connectivity among ROI pairs than Wat controls (AIE - Wat, Fisher’s Z transformed, *p < 0.05, corrected for family-wise error rate). CPu, caudate-putamen; HippD, hippocampus; IL, infralimbic cortex; NAc, nucleus accumbens, OFC, orbitofrontal cortex; PrL, prelimbic cortex; S1, primary somatosensory cortex; Thal, thalamus; AIE, adolescent intermittent ethanol; Wat: water.

We hypothesized that AIE exposure would decrease functional connectivity among regions of interest (ROIs; Figure 5A) that are known to be involved in action selection and learning: PrL, IL, OFC, S1, NAc, CPu, HippD, and Thal. To test this, resting-state fMRI was conducted in approximately half of the rats (*n* = 41); behavioral data on this subset of rats is provided in Supplementary Figures S3–S7. We performed a two-way ANOVA followed by post hoc t-tests to determine the effects of sex and AIE exposure on functional connectivity between the pairs of ROI (Broadwater et al., 2018) and used network-based statistics (NBS) (Zalesky et al., 2010) to control the family-wise error rate. AIE exposure significantly reduced functional connectivity (*p* = 0.02), but no main effect of sex or exposure-by-sex interaction was observed (*p* > 0.05). Specifically, AIE exposure reduced functional connectivity between NAc and CPu (T = 2.53), NAc and PrL (T = 2.54), NAc and S1 (T = 2.63), NAc and HippD (T = 2.96), and NAc and Thal (T = 2.42), as well as IL with HippD (T = 2.49), and PrL with Thal (T = 2.28) (Figure 5B). Note that each T-value above indicates that the AIE exposure effect on the connection between two nodes survived the supra-threshold value that set at *p* < 0.05 for a two-tailed t-test using the NBS approach.

### 3.4 Correlation Between Behavior and fMRI

**FIGURE 6:**
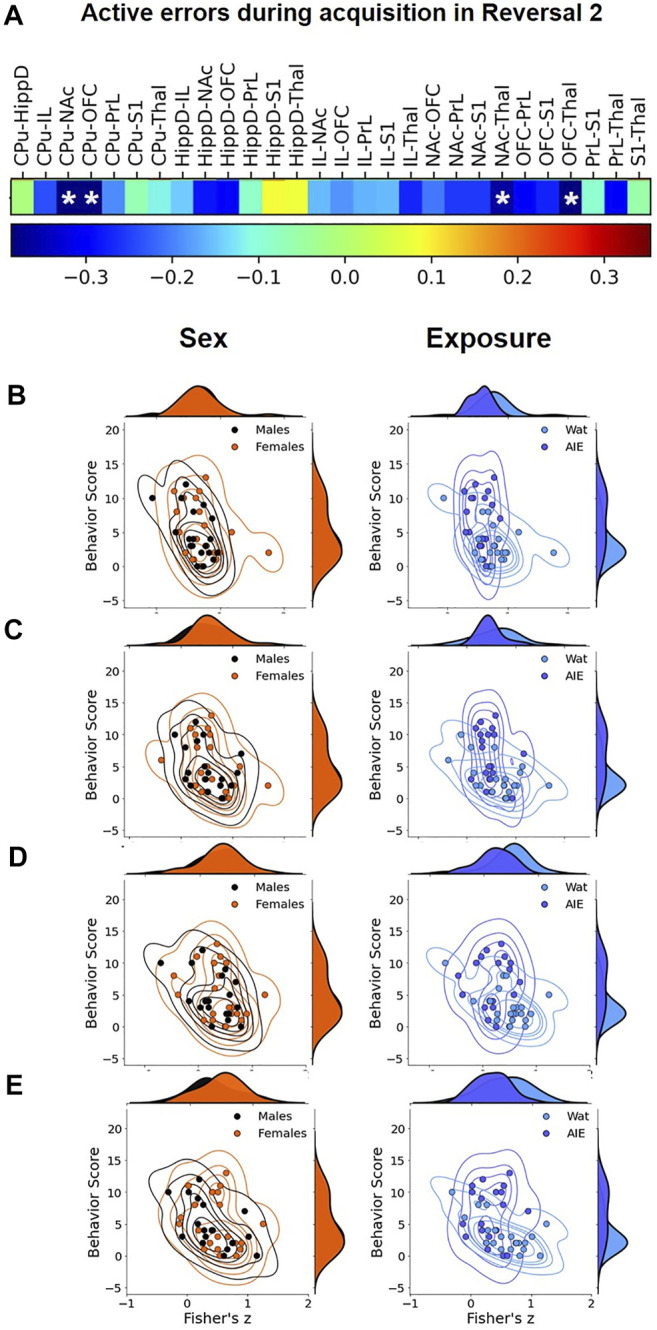
Correlational analysis between active errors during Reversal 2 and functional connectivity among regions of interest (ROIs). We compared the pattern of the values across the subjects between the number of errors in Reversal 2 and pair-wise ROI connectivity to identify the ROI pairs that significantly correlate with behavior at *p* < 0.05. **(A)** In the sequence, we visualized the joint plot for each factor (Sex on left, Exposure on right) to visualize their correlation to the ROI pairs from Panel 5. Most R01 pairs were negatively correlated to active errors in Reversal 2, and four correlations reached statistical significance: **(B)** CPu-NAc; **(C)** CPu-OFC; **(D)** NAc-Thal, and **(E)** OFC-Thal. The data suggest that AIE exposure (right correlation plots) affect the behavior/functional connectivity correlation more than sex (left correlation plots). CPu, caudate-putamen; HippD, hippocampus; IL, infralimbic cortex; NAc, nucleus accumbens, OFC, orbitofrontal cortex; PrL, prelimbic cortex; S1, primary somatosensory cortex; Thal, thalamus; AIE, adolescent intermittent ethanol; Wat: water. Detailed correlational analyses are in Supplemental Table 2.

After identifying exposure effects on functional connectivity, we assessed the relationship between functional connectivity among pairs of ROIs and behavior scores, specifically active errors during acquisition of Reversal 2, a behavioral metric that significantly differed by exposure group (see Table 1 and Supplementary Figure S5). To that end, we performed correlation analysis between behavioral data and connectivity values of each ROI pair (Figure 6A and Supplementary Table S2). The results showed that the functional connectivity of CPu-NAc, CPu-OFC, NAc-Thal, and OFC-Thal pairs showed significant negative correlations with the number of active errors during Reversal 2 acquisition (Figures 6B–E; all Pearson’s R < -0.35, all *p* < 0.05). Scatter plots were used to confirm whether this correlation depended on the experimental factors. As observed in Figures 6B–E, AIE exposure altered the negative correlation identified in water-exposed rats (right columns) with no effect of sex (Figure 6, left columns). Together, our results suggest that AIE exposure disrupted the relationship between ROI connectivity and active errors relative to control animals.

### 3.5 Mediation Analyses

**FIGURE 7:**
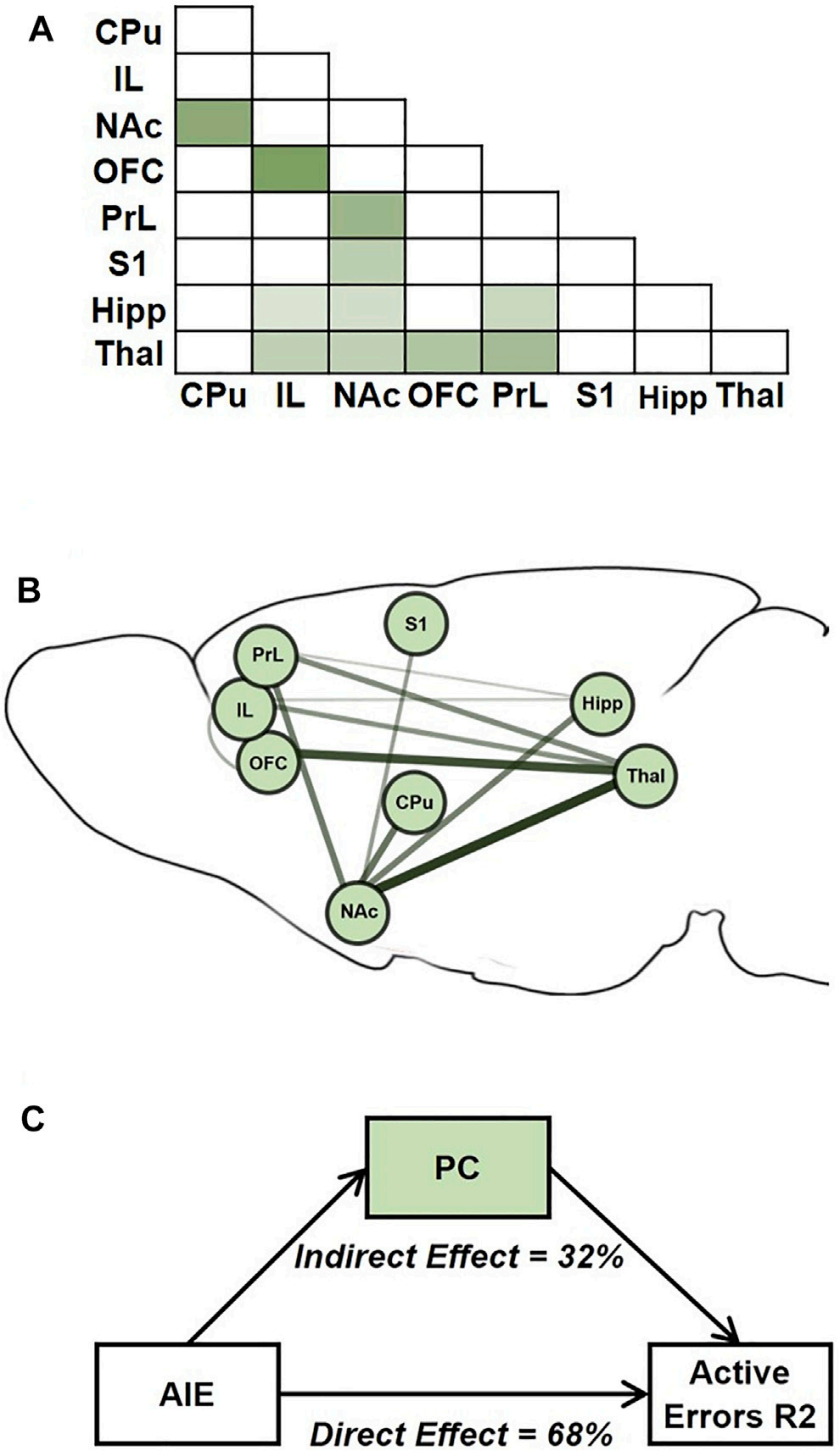
AIE-related changes in brain functional connectivity mediated AIE exposure effects on active errors during Reversal 2. **(A)** Average correlation matrix representing functional connectivity between regions-of-interest (ROIs) for the 11 connections which demonstrated an effect of exposure (*p* < 0.1) and were included in the subsequent subnetwork analysis. Darker color corresponds to stronger functional connectivity. **(B)** A singular value decomposition produced a principal component that explained 66% of shared variance among the 11 connections. The subnetwork identified by the principal component is depicted, with the size and darkness of lines connecting ROIs pairs indicating component weights. **C.** In the mediation analysis, the principal component subnetwork of functional connections accounted for 32% of the effect of exposure on behavior that was explained by the indirect effect through network functional connectivity is indicated. CPu, caudate-putamen; HippD, hippocampus; IL, infralimbic cortex; NAc, nucleus accumbens, OFC, orbitofrontal cortex; PrL, prelimbic cortex; S1, primary somatosensory cortex; Thal, thalamus; AIE, adolescent intermittent ethanol.

While neuroimaging studies in animal models often have the general goal of generating testable hypotheses for direct interventions (e.g., lesion, chemogenetic, or optogenetic studies), a second important use of such studies is for translational comparisons, especially to data from human subjects. Primarily in support of the latter goal, we next tested whether AIE exposure effects on brain connectivity mediated AIE exposure effects on performance in the attentional set-shift task. To do so, we conducted mediation analyses (Figure 7), a statistical method to assess whether inclusion of a mediating variable (*M*) significantly alters the slope of the relationship between two other variables (*X* and *Y*) (MacKinnon et al., 2007; Yung et al., 2018). In this case, *X* represents AIE exposure, *Y* represents set-shift task performance, and *M* was a measure of pairwise connectivity between ROIs. We identified a principal component that reflects a “subnetwork” of functional connections that were most affected by AIE exposure (Figure 7A), and each connection contributed a different weight to the principal component-derived subnetwork (Figure 7B). Of note, this subnetwork included three of the four connections that were independently associated with errors during Reversal 2 based on univariate correlations (i.e., CPu-NAc, NAc-Thal, and OFC-Thal; Figure 6). Next, we assessed whether AIE exposure effects on this functional subnetwork mediated the effects of AIE exposure on the number of errors committed during Reversal 2. The analysis indicated that AIE exposure effects on active errors during Reversal 2 were significantly mediated by brain functional connectivity (*p* < 0.001; Figure 7C). Moreover, functional connectivity in the principal component subnetwork accounted for 32% of the total effect of AIE exposure on active errors during Reversal 2 (*p* = 0.03). Thus, AIE-induced reductions in functional connectivity across brain regions involved in action selection and learning meaningfully contributed to the observed behavioral flexibility deficits.

## 4 Discussion

Behavioral flexibility is a complex construct requiring several constituent functions that recruit multiple brain circuits. Thus, to investigate neural contributions to behavioral flexibility, we chose a network analysis approach over methods that focus on discrete brain regions. While prior studies reported that AIE exposure induces deficits in behavioral flexibility in adult rodents (Coleman et al., 2014; Gass et al., 2014; Madayag et al., 2017; Sey et al., 2019), the behavioral deficits could not be linked to changes in brain circuit function. In this study, we investigated AIE exposure effects on conditioned responding to reward-associated cues (Pavlovian conditioned approach), flexible decision-making (attentional set-shifting task), and resting-state functional connectivity MRI in the same animals to allow direct linkage of functional connectivity among brain regions involved in action selection to behavioral deficits. We did not observe AIE exposure effects on conditioned approach in this study, we did observe clear deficits in reversal learning and a marked reduction in functional connectivity among ROI. We also found a clear association between functional connectivity measures and behavioral performance. Moreover, we identified a functional connectivity subnetwork that mediated reversal learning deficits, demonstrating statistical causation between the degree of functional connectivity and behavioral flexibility. These complimentary analyses identified especially strong contributions of the NAc, thalamus, and OFC in the AIE-induced deficits in behavioral flexibility, a circuit associated with compulsive and inflexible behavior in substance use disorders (Volkow, 2000).

Behaviorally, we observed clear effects of AIE exposure on attentional set-shifting performance. It is worth noting that all observed deficits were identified after ∼55 days from the last alcohol administration and anteceded by PCA training. When considering various phases of the attentional set-shift task, phases implying new learning (e.g., initial acquisition) were not affected by AIE exposure, consistent with previous findings (Risher et al., 2013; Gass et al., 2014), showing that skills required to associate and discriminate stimuli are spared after AIE exposure. However, reacquisition was affected by AIE exposure, even though rats achieved the performance criterion the day before, suggesting deficits in consolidation. Before Reversal 1, AIE exposure impacted reacquisition differently by sex, with AIE-exposed females exhibiting more errors than control females, although the post-hoc comparisons did not reach significance. A potential limitation of this and other aspects of the attentional set task is that we used six consecutive correct choices as the performance criterion. While this criterion allowed us to directly compare our current results with previous reports (Birrell and Brown, 2000; Sey et al., 2019), it is possible that a more stringent criterion would reveal additional behavioral deficits.

We also observed AIE-induced deficits in both males and females during reacquisition before Reversal 2, indicating that although the new rule of Reversal 1 was learned, it was not as effectively consolidated among AIE-exposed animals compared to controls. As AIE-induced impairments in reacquisition performance were more robust before Reversal 2 (that assessed the reversed contingency) than before Reversal 1 (that assessed the original contingency), it is possible that learning of the original contingency was better consolidated in AIE-exposed rats than the reversed contingency. This interpretation is supported by the finding that AIE exposure did not alter reacquisition before the Extradimensional Set-Shift, which also assessed the original contingency. These results are only in partial agreement with our prior study (Sey et al., 2019), in which AIE exposure did not significantly impact any reacquisition phase. Reacquisition is not typically reported (Gass et al., 2014; Fernandez and Savage, 2017; Galaj et al., 2019), and we are not aware of studies evaluating the effect of AIE exposure on memory consolidation. However, AIE exposure alters adolescent hippocampal development (Broadwater et al., 2014; Vetreno and Crews, 2015), a critical region involved in memory consolidation through interaction with other cortical and subcortical regions (see McIntyre et al., 2011 for review).

The introduction of reversal phases revealed interesting differences among AIE-exposed and control groups. In Reversal 1, we observed no differences regarding total trials to criterion or errors, although prepotent responses were marginally different in that AIE-exposed subjects required more trials to adapt to the new rule. In Reversal 2, AIE-exposed rats made more active errors, and specifically more prepotent responses and subsequent regressive errors, while learning the new rule. Thus, AIE-exposed rats were slower to incorporate negative feedback and initiate a new strategy (Buisman-Pijlman et al., 2014) in both reversals, but this effect was stronger in Reversal 2. In contrast, in our previous study (Sey et al., 2019), AIE-induced deficits were observed in Reversal 1, while AIE-exposed rats performed better in Reversal 2. This pattern of behavior indicated that the original attentional set dominated behavioral choice, slowing the ability to learn an initial reversal but facilitating return to that contingency in Reversal 2. A major difference is that in the present study, rats had Pavlovian conditioning training prior to assessing attentional set learning, and previous behavioral training can impact subsequent task performance (Klintsova et al., 2002; Hannan, 2014; Fernandez and Savage, 2017). However, both studies identified increased prepotent responses in the AIE-exposed groups, consistent with perseveration on the previous response despite negative feedback. This particular deficit is suggestive of impaired inhibitory control (Luna, 2009; Adele; Diamond, 2013) and is consistent with other studies using AIE (Coleman et al., 2014; Fernandez and Savage, 2017; Galaj et al., 2019) and chronic intermittent ethanol (CIE) exposure in adult rodents (Badanich et al., 2011), and even in humans (for a recent review see López-Caneda et al., 2014). Moreover, the present study found that subsequent regressive errors were higher after AIE exposure. In general, regressive errors suggest a lack of ability to maintain a new association or keep the current rule “in mind” despite feedback.

Thus, our results suggest an AIE-induced deficit in working memory, a conclusion supported by other studies showing adolescent alcohol effects on that function in animals (Schulteis et al., 2008; Salling et al., 2018; Macht et al., 2020) and, less consistently, in humans (Salas-Gomez et al., 2016; Mahedy et al., 2018). Overall, these reversal data suggest that AIE exposure induces deficits in the fundamental processes of inhibitory control and working memory, resulting in deficits in flexible decision-making. Future studies can directly assess inhibitory control and working memory in animals with and without AIE exposure to confirm this interpretation.

Perseveration of an acquired attentional set illustrates how well-learned cues can retain control over action selection after AIE exposure. However, perseveration did not extend to the sensory domain of the relevant cue: when novel odor and tactile stimuli were used in the extradimensional set shift, all rats readily learned to pay attention to the tactile rather than the odor cue, consistent with previous studies (Badanich et al., 2011; Sey et al., 2019) using the digging task to assess alcohol effects on attentional sets. In contrast, studies using an operant-chamber version of a set-shift from a visual rule to a location rule reported AIE-induced deficits after chronic alcohol exposure (Hu et al., 2015) and AIE (Gass et al., 2014; Fernandez and Savage, 2017) [with one study only observing an AIE effect in males (Varlinskaya et al., 2020)]. However, in this latter task, the formerly relevant cues remain in the chamber, and the subject must inhibit responses to the visual cues and respond instead by location. Thus, those data can also be interpreted as an inability to inhibit a previously learned attentional set in AIE-exposed animals, as opposed to inability to shift the relevant stimulus from one domain to another.

While we replicated a sex difference in sign-tracking behavior in Pavlovian conditioned approach (Madayag et al., 2017), we did not observe an effect of AIE exposure on conditioned approach in the present study, in contrast to other reports in the literature (McClory and Spear, 2014; Kruse et al., 2017; Madayag et al., 2017). Large individual differences are often observed in conditioned approach due to considerable variability among animals in the propensity to attribute incentive salience to cues (Flagel et al., 2007; Robinson et al., 2014). In addition, there is large variability in the field around Pavlovian conditioning methodology, such as the timing of the cue and the length of training, and these factors may have contributed to our outcome. Similar issues have arisen in the literature reporting “attentional-bias” to appetitive cues in humans, a behavior that is likened to Pavlovian conditioning. Variations in the methods used to evaluate attentional bias, such as task psychometric properties, sample size, or cue sensory modality, among others, potentially explain inconsistent results among studies (Cisler et al., 2010; Field et al., 2010). While we did not replicate our previous findings, the majority of studies (McClory and Spear, 2014; Kruse et al., 2017; Madayag et al., 2017) to date have reported shifts toward sign-tracking after AIE exposure, suggesting enhanced sensitivity to reward-associated cues or incentive salience attribution. It’s worth noting that we did not test the persistence of conditioned approach behavior (e.g., via reward omission) or the development of incentive salience of the conditioned cue (e.g., via Pavlovian instrumental transfer). Thus, an empirical question is whether flexibility in these aspects of reward conditioning is altered by AIE exposure.

Action selection and behavioral flexibility in response to changing circumstances are largely mediated by cortico-striatal-thalamic circuits (McKim et al., 2016). Moreover, gaps in memory encoding or consolidation might be associated with reduced function of prefrontal and hippocampal areas (Carbia et al., 2018; Takehara-Nishiuchi, 2020), deficits in inhibitory control with fronto-striatal regions (Diamond, 2013; López-Caneda et al., 2014), and impairments in working memory and behavioral flexibility mainly with prefrontal regions (Diamond, 2013; Crews et al., 2019)—all brain regions that have been shown to be affected by AIE exposure (Crews et al., 2019). Thus, we hypothesized that behavioral deficits would be associated with altered functional connectivity among fronto-limbic regions in AIE-exposed animals. We found that AIE exposure reduced functional connectivity across multiple ROI pairs, including frontal, hippocampal and thalamic connections with the NAc. We previously observed similar AIE-induced reductions in functional connectivity across fronto-striatal regions of male rats (Broadwater et al., 2018), and the present study extended those findings to females. Indeed, the present findings suggest a robust disruption of NAc functional connectivity. Independent studies have shown the crucial role of this brain region in behavioral flexibility (Radke et al., 2019; Korn et al., 2021), and other studies have reported effects of AIE exposure on NAc physiology (Spoelder et al., 2015; Shnitko et al., 2016). Thus, a major contribution of the current study was to determine multiple specific connections with the NAc that were statistically linked to behavioral flexibility as well as affected by AIE exposure.

Linking the MRI data to behavioral assessments, the correlational analysis between pairwise connectivity strength and behavioral performance in Reversal 2 showed a negative association between functional connectivity in specific ROI pairs (including OFC, NAc, CPu, and Thal) and observed behavioral deficits. Interestingly, the correlation was stronger in water-exposed rats and reduced in AIE-exposed rats, suggesting that ethanol disrupted the association between functional connectivity and the behavior. Since we were underpowered to statistically validate this statement, additional studies including more animals can clarify this effect. An additional aspect derived from this analysis was the alleged key role that connections between OFC, NAc, and Thal play in the effects of AIE exposure on behavioral deficits, something also observed in the mediation analysis.

To address the interconnected nature of the ROIs, we used principal component analysis of functional connections to identify a principal component or “subnetwork” that might mediate observed behavioral deficits. The functional connections of the identified subnetwork (accounting for 66% of variance) include brain regions and projections observed in several studies to be involved in the cognitive processes of flexible decision-making (Jongen-Rêlo et al., 2003; Seamans et al., 2008; Yoon et al., 2008; Luna, 2009; Moore et al., 2013; Wang et al., 2015; Davies et al., 2017; Hardung et al., 2017; Esmaeili and Diamond, 2019). Interestingly, two connections that contributed most strongly to this subnetwork—NAc-Thal and Thal-OFC—form the striato-thalamo-orbitofrontal circuit that is hypothesized to be persistently impaired after repeated exposure to drugs of abuse and to contribute to the compulsive and inflexible behaviors associated with addiction (Volkow, 2000). The finding that adolescent alcohol exposure, in the absence of “addiction,” can affect similar circuits as those affected by chronic substance use supports an apparent vulnerability of prefrontal structures implicated in “top-down” subcortical regulation to drug exposure (Volkow, 2000; Spear, 2018; Crews et al., 2019).

Using this subnetwork as a mediator, we determined that AIE-associated decreases in functional connectivity mediated the increases in active errors during Reversal 2, when both contingencies had been experienced. While at least one prior study has correlated specific MRI measures to animal behavior (Li et al., 2018), to our knowledge, this is the first study to demonstrate that changes in functional connectivity statistically mediated behavioral performance in the same set of animals. Thus, it is validating that the different approaches to assess the impact of AIE exposure on brain functional connectivity yielded converging results highlighting connections between prefrontal, accumbal and thalamic regions. These findings also support future studies to explore individual pathways identified in the present data as relevant to cognitive flexibility (NAc-Thal and Thal-OFC) and its involvement in behavioral deficits after adolescent alcohol exposure.

There are many possible mechanisms that may underlie this altered functional connectivity following adolescent alcohol exposure, one of which is AIE-induced changes in cholinergic function. There is compelling evidence showing decreases in forebrain cholinergic phenotypic markers (Ehlers et al., 2011; Coleman et al., 2014; Fernandez and Savage, 2017; Galaj et al., 2019) and receptors (Coleman et al., 2014) after AIE exposure in rodents, supporting AIE-induced reductions in cholinergic neurotransmission that persist into adulthood and likely affect behavior (see Crews et al., 2019 for a review). Basal forebrain cholinergic neurons project extensively throughout the brain, including several regions in the functional connectivity subnetwork we identified: prefrontal cortex, hippocampus, and sensory cortex receive direct, modulatory cholinergic projections from the basal forebrain (Picciotto et al., 2012; Muñoz and Rudy, 2014). Documented cholinergic involvement in cognitive processes like learning, attention and memory [including working memory (Ballinger et al., 2016; Eckart et al., 2016)] implicates acetylcholine as a crucial player in cognitive performance. Thus, changes in cholinergic dynamics might explain behavioral and connectivity impairments in AIE-exposed animals; however, this hypothesis remains to be directly tested.

A strength of this study is large number of animals included, particularly for the behavioral measurements, which allowed for assessment of sex as a biological variable. Although no statistical differences were observed in functional connectivity among males and females, we found that females displayed a stronger deficit in reversal learning after AIE exposure and more sign-tracking behavior relative to males. While a sex difference in sign-tracking behavior is consistent with the literature (Pitchers et al., 2015; Sey et al., 2019; Stringfield et al., 2019), many studies assessing reversal learning after AIE exposure have only investigated cognitive deficits in males (Gass et al., 2014), were not sufficiently powered to address sex differences (Sey et al., 2019), or presented similar AIE effects between males and females (Galaj et al., 2019). However, understanding the underlying mechanisms that are sex-specific, such as immune reactivity and maturation of neural networks (Bilbo, 2018), may explain the domain, severity and persistence of cognitive deficits in females versus males.

It is important to note that animals were “passively” exposed to alcohol via experimenter-delivered gavage through a widely used protocol designed by the NADIA consortium (Crews et al., 2019) and tested in our lab (Madayag et al., 2017; Sey et al., 2019) and others (Vetreno and Crews, 2015; Dannenhoffer et al., 2018). This protocol ensures binge-like blood ethanol concentrations, modeling intermittent binge drinking that is often observed in human adolescents who drink. While beyond the aims of this study, it is possible that different results would be obtained if rats consumed the ethanol, as some behavioral and neurochemical differences have been observed between experimenter-delivered and self-administered alcohol (e.g., Weise-Kelly and Siegel, 2001; Orrù et al., 2016). A limitation of the present study is that animals were sedated during MRI scans rather than awake. While awake rat fMRI is possible, we used the most widely used light anesthesia protocol (Fukuda et al., 2013) to avoid the potential confounds of habituation and restraint after our behavioral approaches. Importantly, studies have shown that major brain connectivity changes are preserved across anesthesia states (Ma et al., 2017). Finally, while this study documented AIE-induced changes in behavioral flexibility and network connectivity several weeks after ethanol exposure, future studies can determine whether these effects of ethanol are specific to adolescent exposure and how they change across the lifespan.

In summary, we identified a functional connectivity subnetwork based on selected ROIs that was highly predictive of behavioral performance and mediated the effect of AIE exposure on reversal learning. This also suggests an effect of adolescent alcohol exposure on the striato-thalamo-orbitofrontal circuit, which has been related to compulsive behavior and risk for developing substance use disorders (Volkow, 2000). Here, we provided support for the hypothesis that decreased functional connectivity after AIE exposure mediates deficits in cognitive processing that impair flexible decision-making. Moreover, by using a network analysis approach, we provide evidence about AIE effects on the functional system that underlies complex processes involved in flexible decision-making, adding a fundamental piece for the understanding of binge-drinking effects on brain development and function.

## Significance Statement

Alcohol binge drinking among adolescents is a highly prevalent phenomenon in the USA, and current research has shown that this behavior impacts brain function and cognition. However, most evidence comes from independent studies, limiting their ability to link the neural and behavioral effects of alcohol. Here, we evaluated persistent alcohol effects on brain function and behavior in the same set of animals. We demonstrated that adolescent alcohol exposure reduced both behavioral flexibility and functional connectivity among key brain regions. For the first time, we reported that alcohol-induced reductions in functional connectivity mediated a large proportion of observed behavioral deficits. This evidence reinforces the premise that adolescent alcohol exposure can have long-lasting effects on brain function and associated behavior and cognition, highlighting the importance of strategies to prevent adolescent binge drinking.

## Data Availability

The raw data supporting the conclusion of this article will be made available by the authors, without undue reservation.
